# The Genetic Cross-Talk between Periodontitis and Chronic Kidney Failure Revealed by Transcriptomic Analysis

**DOI:** 10.3390/genes14071374

**Published:** 2023-06-28

**Authors:** Dandan Ren, Thomas Ebert, Deborah Kreher, Bero Luke Vincent Ernst, Jonathan de Fallois, Gerhard Schmalz

**Affiliations:** 1 School of Pharmacy, Nanjing Medical University, Nanjing 211166, China; rendandan96@njmu.edu.cn; 2 Medical Department III—Endocrinology, Nephrology, Rheumatology, University of Leipzig, 04013 Leipzig, Germany; thomas.ebert@medizin.uni-leipzig.de (T.E.); jonathan.defallois@medizin.uni-leipzig.de (J.d.F.); 3 Department of Cariology, Endodontology and Periodontology, University of Leipzig, 04103 Leipzig, Germany; deborah.kreher@medizin.uni-leipzig.de (D.K.); bero.ernst@medizin.uni-leipzig.de (B.L.V.E.)

**Keywords:** kidney failure, periodontitis, inflammation, bioinformatics

## Abstract

Periodontitis and chronic kidney failure (CKF) are potentially related to each other. This bioinformatics analysis aimed at the identification of potential cross-talk genes and related pathways between periodontitis and CKF. Based on NCBI Gene Expression Omnibus (GEO), datasets *GSE10334*, *GSE16134*, and *GSE23586* were extracted for periodontitis. A differential expression analysis (*p* < 0.05, |log2(FC)| > 0.5) was performed to assess deregulated genes (*DEGs*). CKF-related genes were extracted from DisGeNET and examined regarding their overlap with periodontitis-related *DEGs*. Cytoscape was used to construct and analyze a protein–protein interaction (*PPI*) network. Based on Cytoscape plugin MCODE and a LASSO regression analysis, the potential hub cross-talk genes were identified. Finally, a complex *PPI* of the hub genes was constructed. A total of 489 *DEGs* for periodontitis were revealed. With the 805 CKF-related genes, an overlap of 47 cross-talk genes was found. The *PPI* network of the potential cross-talk genes was composed of 1081 nodes and 1191 edges. The analysis with MCODE resulted in 10 potential hub genes, while the LASSO regression resulted in 22. Finally, five hub cross-talk genes, *CCL5*, *FCGR3B*, *MMP-9*, *SAA1*, and *SELL*, were identified. Those genes were significantly upregulated in diseased samples compared to controls (*p* ≤ 0.01). Furthermore, ROC analysis showed a high predictive value of those genes (AUC ≥ 73.44%). Potentially relevant processes and pathways were primarily related to inflammation, metabolism, and cardiovascular issues. In conclusion, five hub cross-talk genes, i.e., *CCL5*, *FCGR3B*, *MMP-9*, *SAA1*, and *SELL*, could be involved in the interplay between periodontitis and CKF, whereby primarily inflammation, metabolic, and vascular issues appear to be of relevance.

## 1. Introduction

Periodontal diseases are highly prevalent inflammatory changes in the tissues surrounding the teeth; while a biofilm association is evident, the multifactorial etiology of periodontitis is an issue of increasing interest [1]. As such, periodontal diseases are closely related with systemic inflammation and thus with a variety of systemic diseases, including diabetes mellitus, rheumatic diseases, or atherosclerosis [2]. In this respect, an association between periodontitis and chronic kidney diseases appears evident, too; a recent meta-analysis concluded periodontitis to be a frequent co-morbidity of chronic kidney diseases [3]. 

It is documented that co-morbidities, especially diabetes mellitus, are a potential cause for the joint occurrence of periodontitis and kidney diseases [4,5]. Additionally, reduced oral health behavior and a decreased perception of oral health concerns, resulting in low utilization of dental health care, have been discussed to explain a relationship between periodontitis and kidney disease, especially in the end stage of kidney failure [6,7]. However, the recent literature discusses more and more a causal, potentially bidirectional relationship between both diseases [5,8,9]. As such, microbiological effects, originating from potentially periodontal pathogenic bacteria, which have the ability to translocate and have distal effects outside of the periodontal pocket, have been discussed [8,10]. Another relevant factor potentially linking periodontitis and kidney diseases is the role of cytokines and oxidative stress [8]. The periodontitis-related increase in pro-inflammatory cytokines can also lead to an elevated systemic inflammation, potentially supporting renal atherosclerosis, renal deterioration, and end-stage renal disease development [8]. Another approach to explain the causality between periodontal and kidney diseases was an examination of genetic variables, especially single-nucleotide polymorphisms, whereby no causal relationship between both diseases has yet been confirmed [9]. However, the relationship between periodontitis and outcome of patients with kidney diseases, especially the risk of arteriosclerosis [11] or even mortality [12], supports the high relevance of this interrelationship as an issue of further research. Against this background, studies appear to be required to increase the understanding of the association between periodontitis and kidney diseases, especially chronic kidney failure (CKF). 

One potential approach could be the application of bioinformatics analysis to explore a potential overlap between both diseases on the transcriptomic level. Previously, applied bioinformatics has been used to gain insight into the relationship between periodontitis and oral cancer [13], atherosclerosis [14] or chronic obstructive pulmonal diseases (COPD) [15]. Thereby, a variety of potential cross-talk genes for those diseases, related pathways, and biological processes were revealed, which can form a reasonable basis for future clinical research in the field. Accordingly, this current study aimed at the evaluation of potential cross-talk genes between periodontitis and CKF by using transcriptomic analysis. Therefore, a complex and comprehensive analysis was performed, including the definition of different risk groups and clusters, as well as a regulatory network of the respective cross-talk genes. It has been hypothesized that periodontitis and CKF would be connected by different cross-talk genes, which would be involved in the regulation of (pro-)inflammatory processes and pathways. 

## 2. Materials and Methods

### 2.1. Datasets

Datasets for human periodontitis (PD)-related gene expression profiles were assessed from the GEO (http://www.ncbi.nlm.nih.gov/ (assessed on 2 November 2022) database. Samples with expression profile characteristic of gingival tissues were selected, and finally 3 PD-related datasets were obtained (*GSE10334*, *GSE16134*, *GSE23586*), which were based on the GPL570 platform. Chronic and aggressive periodontitis samples were allocated to the case group and healthy samples to the control group. Data for chronic kidney failure (CKF)-related genes were searched from DisGeNET (https://www.disgenet.org/home/ (assessed on 4 November 2022), whereby finally 805 CKF-related genes were obtained.

### 2.2. Data Pre-Processing

First, the probe IDs of the three PD datasets (*GSE10334*, *GSE16134*, *GSE23586*) were converted to the corresponding gene names based on the platform information of GPL570. To ensure the uniqueness of the gene names, the mean of the expression values of these probes in a certain sample was assessed as the expression value of the gene in this sample. In addition, since the *GSE23586* expression values were not in the same order of magnitude as the *GSE10334* and *GSE16134* expression values, a log2 scaling on the *GSE23586* expression matrix was performed.

### 2.3. Genes Differentially Expressed in PD (*DEGs*)

Under application of the “limma” package of the R language, differential expression analysis was performed for each of the three PD-related expression matrices. The selection of differentially expressed genes was generally executed according to empirical values. Consequently, selected genes with *p*-value < 0.05, |log2(FC)| > 0.5 were considered as differentially expressed genes (*DEGs*). Based on the results of limma analysis, the up- and downregulated *DEGs* of the three datasets were recorded, along with the genes that were co-upregulated and co-downregulated *DEGs*. Common genes which were significantly different between case and control in all three sets of PD data were included in the subsequent analysis.

### 2.4. Cross-Talk Genes for PD and CKF

*DEGs* of PD were investigated regarding their overlap with CKF-associated genes, whereby overlapping genes, which were significantly deregulated in both diseases, were labeled as potential cross-talk genes. R’s clusterProfiler package was used to perform GO Biological process and KEGG pathway analysis of those cross-talk genes to observe the biological processes affected by these genes.

### 2.5. Cross-Talk Gene *PPI* Network Analysis

The protein–protein interaction (*PPI*) relationship pairs of cross-talk genes were obtained from HPRD (http://www.hprd.org/ (assessed on 5 November 2022) and BIOGRID (http://thebiogrid.org/ (assessed on 5 November 2022) databases. Subsequently, Cytoscape (version 3.9.1) was used to construct the *PPI* network. After the network was constructed, it was analyzed using the topological property analysis function of Cytoscape (https://med.bioinf.mpi-inf.mpg.de/netanalyzer/help/2.7/index.html#complex (assessed on 5 November 2022). Finally, a module mining of the network was performed using the Cytoscape plugin MCODE to filter the hub genes in the module (MCODE), which should be included in the subsequent analysis.

### 2.6. GSVA (Gene Set Variation Analysis) Analysis

Datasets of pathways and genes were assessed from Molecular Signatures Database (MSigDB, https://www.gsea-msigdb.org/gsea/msigdb/ assessed on 6 November 2022). Then, the three datasets, *GSE10334*, *GSE16134*, and *GSE23586*, were combined based on common genes, considering all samples together to form the PD expression matrix. Afterwards, the expression values of *DEGs* and CKF-related genes in PD were evaluated to perform GSVA for enrichment analysis of the expression profiles of PD- and CKF-related genes in PD based on the pathway and gene datasets, respectively. Based on the case and control groups, a differential expression analysis of GSVA results was performed using the limma package of R (comparison case vs. control). Based on the results of differential expression analysis, the pathways with |log FC| > median (|log FC|), *P*.adjust < 0.05 were assessed as significant difference-related pathways. After obtaining the pathways with significant differences between PD and CKF, the two intersecting pathways were selected for inclusion in the subsequent analysis.

### 2.7. Consensus Cluster Plus Analysis

From the results of GSVA analysis, significant difference intersection pathways of PD and CKF were obtained. The significant difference intersection pathways in PD- and CKF-related genes were evaluated in the PD case sample score matrix. Then, two sets of score matrices were merged and the genes were de-weighted using the mean, finally obtaining the PD&CKF significant difference pathway dataset. Then, the Consensus Cluster Plus algorithm was utilized to perform k-means clustering analysis on the PD&CKF significantly different pathway dataset.

Two methods were applied to reveal the optimal number of clusters: an average silhouette width and elbow method (also called sum of square error, or SSE), to analyze the PD&CKF significantly different pathways dataset. These two methods were applied using the fviz_nbclust method of the R language factoextra package. Average silhouette width automatically calculates the number of optimal clusters. The elbow method requires observing the slope of the vertical coordinate total Within Sum of Square (WSS). When the WWS decreases slowly, increasing the number of clusters can no longer increase the clustering effect, and the “elbow point” is the optimal number of clusters. The results of these two methods were used to determine the optimal number of clusters. Furthermore, the PD&CKF significantly different pathways dataset was analyzed using the ConsensusClusterPlus package in R. Using the Consensus Cluster Plus algorithm, the PD&CKF significantly different pathway dataset was divided into different clusters by case samples. Finally, the Kruskal test was used to detect the significant differences between different clusters of the pathway to see the effect of clustering of samples.

### 2.8. Screening of Hub Cross-Talk Gene

Firstly, an ANOVA analysis was performed on the cross-talk gene expression matrix according to clusters, and the ANOVA analysis results in *p*-value correction to obtain *P*.adjust values, and genes with *P*.adjust < 0.05 were selected for inclusion in the subsequent analysis. Then the features of cross-talk genes after ANOVA analysis were filtered using LASSO (Least absolute shrinkage and selection operator) Logistic Regression.

Two sets of values were obtained in the results of LASSO analysis, i.e., lambda.min and lambda.1se. Lambda.min is the value of lambda that gives the minimum cvm and lambda.1se is the largest value of lambda such that the error is within 1 standard error of the minimum. In this current analysis, the result of lambda.1se was chosen to filter the cross-talk genes. The hub genes obtained from the *PPI* network (MCODE) and the hub genes obtained from the LASSO analysis (LASSO) were assessed. These cross-talk genes were the final screen for hub cross-talk genes, which are potentially associated genes in the disease process in PD and CKF.

### 2.9. Prediction of Cluster Risk Based on Hub Cross-Talk Genes

After obtaining the hub cross-talk genes, their coefficient of regression was assessed in the results of LASSO regression analysis. Based on the hub cross-talk gene expression matrix and coefficient of regression, the case sample risk score was calculated, and the risk score was calculated as Risk score = ∑ 𝑋𝑖 ∗ 𝑌𝑖 (*X* is the regression coefficient and *Y* is the gene expression value). Then, min–max normalization was performed on the risk score to map the risk score to the interval [0,1]. After calculating the sample risk score, the samples were divided into high-risk and low-risk groups based on the median. The distribution of high-risk samples and low-risk samples in different clusters is observed to achieve the prediction of cluster risk.

### 2.10. Hub Cross-Talk Gene in-Depth Analysis

The gene expression values of hub cross-talk genes and non-hub cross-talk genes in the PD dataset were assessed and then analyzed regarding the relationship between hub cross-talk gene and non-hub cross talk gene using Pearson correlation coefficient. Moreover, the relationship between hub cross-talk gene and sample risk score was analyzed. Then, box line plots were used to view hub cross-talk gene expression in case vs. control and high-risk vs. low-risk in cluster 1–3. The Wilcoxon test was used to see the significant relationships between hub cross-talk genes in case vs. control and high-risk vs. low-risk. The Kruskal test was applied to analyze the relationship of the significant hub cross-talk genes in cluster 1–3. Based on case vs. control sample type, ROC analysis was performed on hub cross-talk gene expression values by observing AUC (Area Under Curve) to assess gene expression levels.

### 2.11. Hub Cross-Talk Gene *PPI* and Pathway Network

*PPI* relationship pairs were analyzed between the hub cross-talk genes and other genes from the results of *PPI* network analysis, which we noted as hub gene–target1 relationship pairs. Based on the KEGG database (https://www.kegg.jp/ (assessed on 8 November 2022), the hub gene–pathway relationship pairs were assessed. Based on the pathways in the hub gene–pathway relationship pairs, the genes under these pathways were extracted from KEGG, so the pathway–target2 relationship pairs were obtained. Hub gene–target1 relationship pairs, hub gene–pathway relationship pairs, and pathway–target2 relationship pairs were merged to construct hub gene–pathway–target2 composite relationship pairs. Then, the genes in target1 were screened from the target2 gene set to form the target1–hub gene–pathway–target1 by closed-loop relationship chain. Finally, Cytoscape was used to construct the target1–hub gene–pathway–target1 closed-loop structural network.

## 3. Results

### 3.1. DEG in PD

**Figure 1:**
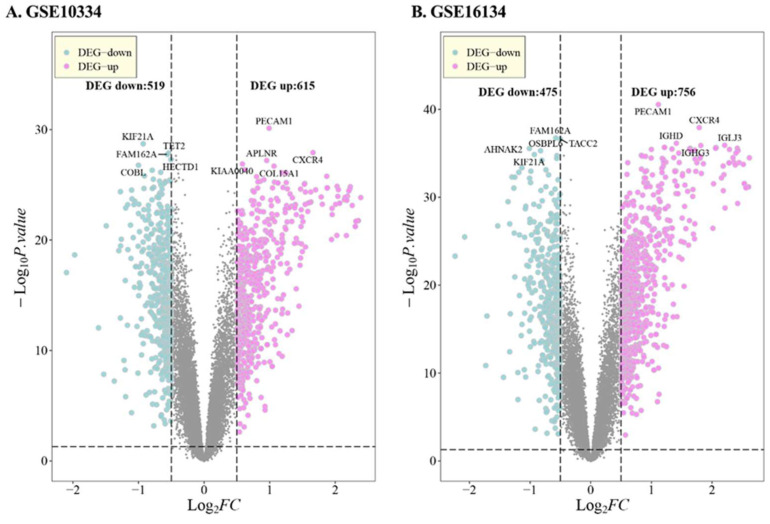

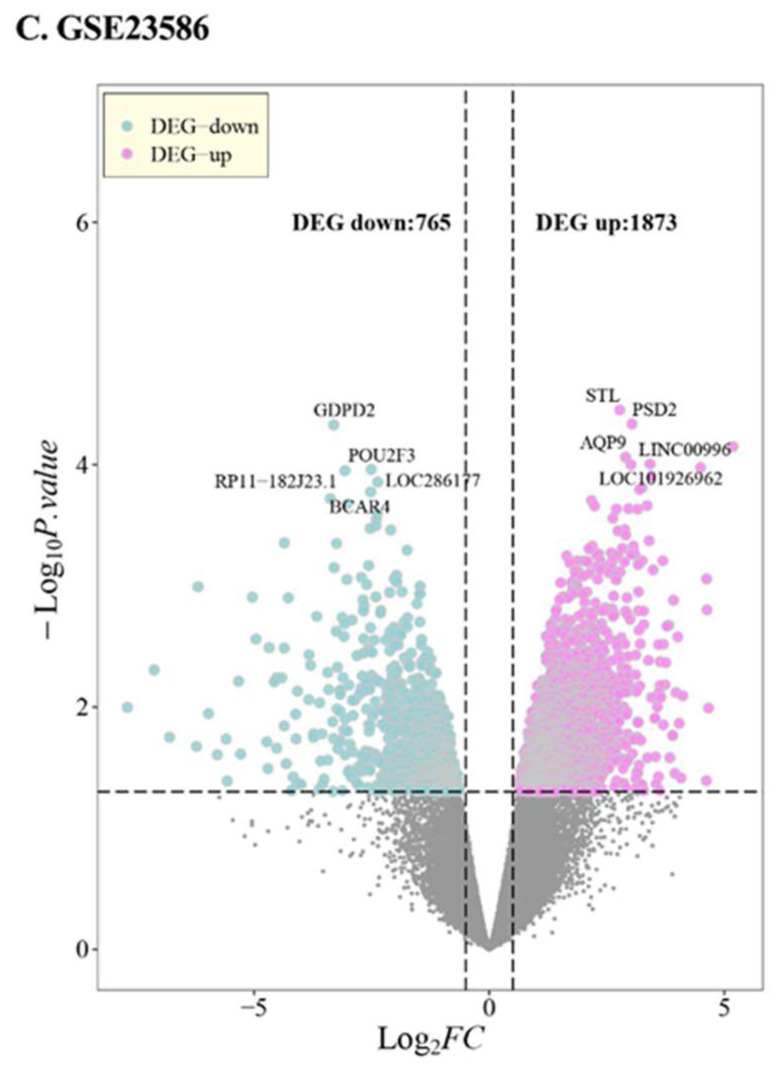
Differential expression analysis. Top5 of DEG-up and DEG-down significant genes are marked in the figure, respectively. (**A**) shows the expression in *GSE10334*, (**B**) in *GSE16134* and (**C**) in *GSE23586* datasets.

**Figure 2:**
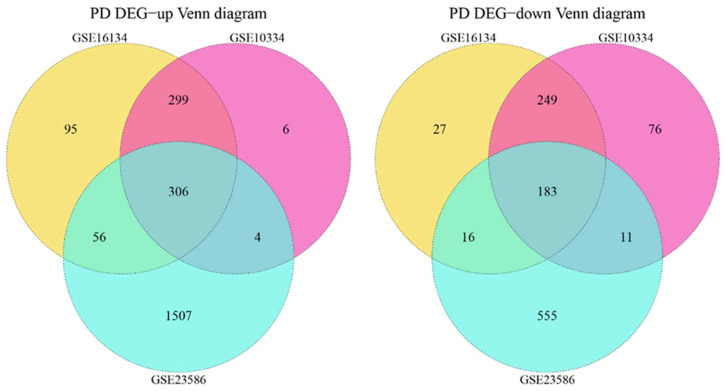
Venn diagram of the relationship between *DEGs* of PD-related datasets (*GSE10334*, *GSE16134*, and *GSE23586*).

Genes with *p*-value < 0.05, |log2(FC)| > 0.5 were seen as differentially expressed genes, where Log2(FC) > 0.5 was upregulated and log2(FC) < −0.5 was downregulated. The results of the difference analysis are shown in Table 1. Figure 1 illustrates a volcano map, showing the regions where the datasets differed in genes, whereby the significant Top5 genes in different regulatory genes are depicted. Taking the intersection of *DEGs* of *GSE10334*, *GSE16134*, and *GSE23586*, a total of 489 PD-associated *DEGs* were obtained, of which 306 were co-upregulated and 183 were co-downregulated (Figure 2).

### 3.2. Cross-Talk Gene Analysis

**Figure 3:**
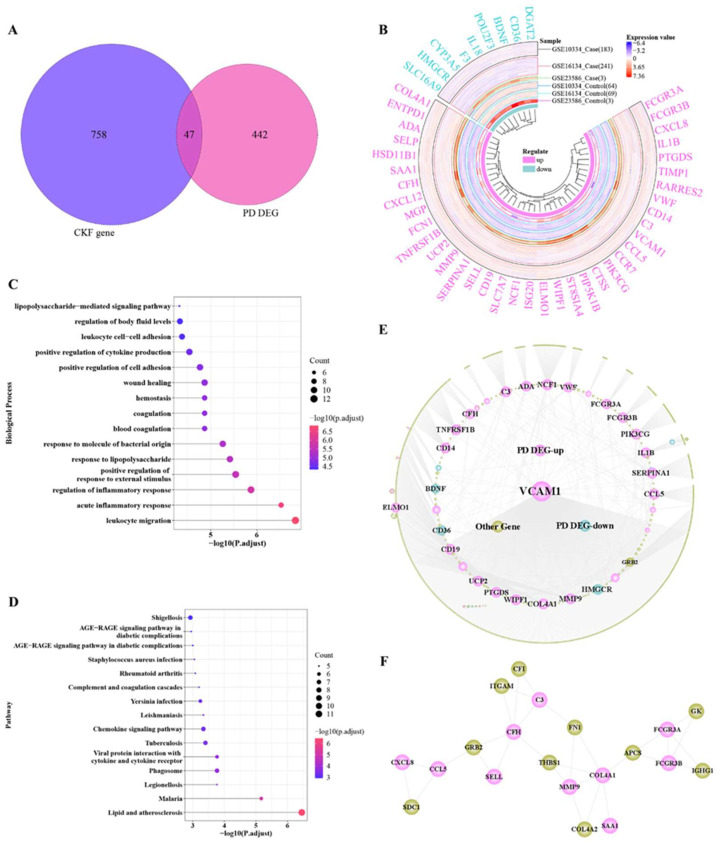
Cross-talk gene function and *PPI* network analysis. (**A**) Venn diagram of PD differentially expressed genes and CKF-associated genes, whereby the overlap region represents the cross-talk genes; (**B**) expression trends of cross-talk genes in PD datasets (*GSE10334*, *GSE16134*, and *GSE23586*); (**C**) cross-talk genes involved in biological processes; (**D**) pathways regulated by cross-talk genes; (**E**) *PPI* network associated with cross-talk genes; (**F**) modules associated with cross-talk genes.

Combining the 489 PD-associated *DEGs* and 805 CKF-associated genes (Figure 3A), 47 cross-talk genes were commonly associated with PD and CKF. Table 2 summarizes the results of differential expression analysis of those 47 cross-talk genes in *GSE10334*, *GSE16134*, and *GSE23586*.

After the extraction of the expression values of 47 cross-talk genes, R’s circlize package and ComplexHeatmap package were used to show the differences between the expression levels of cross-talk genes in different sample groups (Figure 3B). To better observe the differences between case and control samples, the data used for drawing the map were normalized for all sample expression values under each gene. 

GO Biological process and KEGG pathway analysis on these 47 cross-talk genes revealed a couple of significant pathways; Top15 pathways are shown in Figure 3C,D. A total of 45 proteins with interactions in the cross-talk genes were obtained from *PPI* data. The cytoscape-based cross-talk gene-related *PPI* network is shown in Figure 3E. The *PPI* network is composed of 1081 nodes and 1191 edges, where 1081 nodes contain 36 upregulated cross-talk genes, 9 downregulated cross-talk genes, and 1036 other genes. The results of topological property analysis of 45 cross-talk genes are illustrated in Table 3. From Table 3 and Figure 3E, it can be seen that genes such as VCAM1, NCF1, HMGCR, CD36 are highly connected in the network. The network modules were mined using MCODE plugin and one module (Figure 3F) was obtained. The module is composed of 10 hub genes, i.e., *COL4A1*, *SELL*, *FCGR3A*, *CXCL8*, *MMP9*, *SAA1*, *CFH*, *CCL5*, *FCGR3B*, and *C3*.

### 3.3. GSVA Analysis

**Figure 4:**
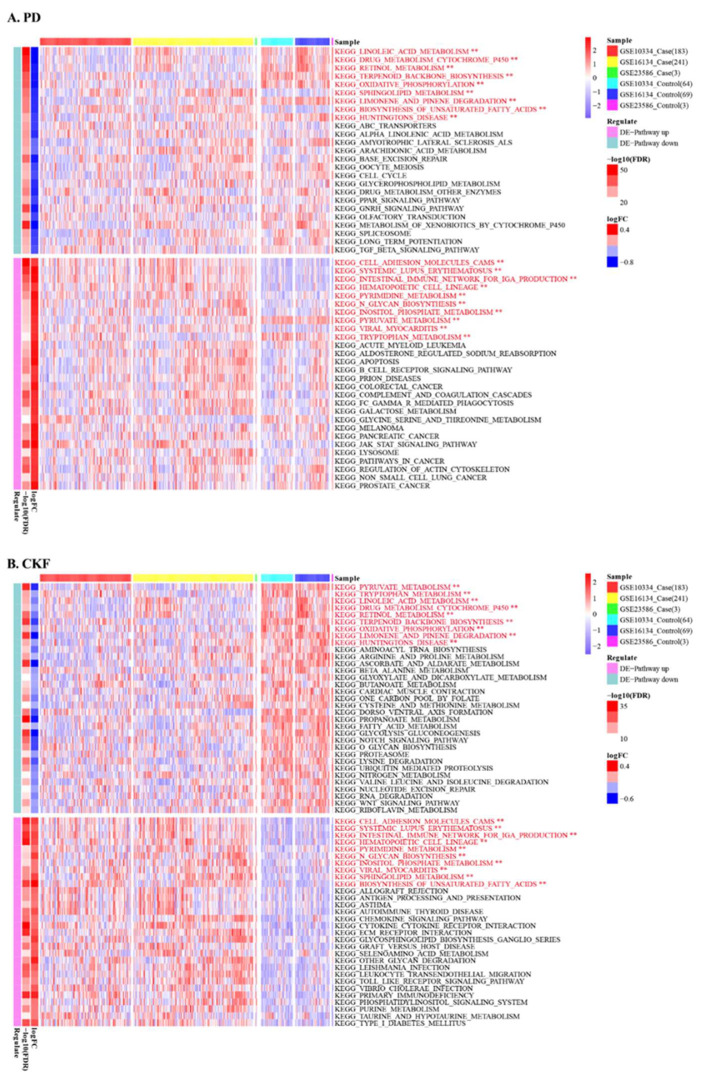
Results of GSVA enrichment analysis of PD- and CKF-related genes in PD expression profiles (** statistically significant).

All samples in *GSE10334*, *GSE16134*, and *GSE23586* were merged, resulting in a PD expression matrix consisting of 427 case and 136 control samples. From there, 489 PD-associated *DEGs* and 805 CKF-associated genes were extracted from the expression matrices, and these were the basis for GSVA analysis on the two sets of expression matrices (Figure 4). The results of limma analysis based on PD-related *DEGs* had median (|log FC|) = 0.413, so in PD log2FC < 0.413 was considered a differentially upregulated pathway and log2FC < −0.413 a differentially downregulated pathway. Based on the results of limma analysis of CKF-related genes in median (|log FC|) = 0.228, CKF log2FC < 0.228 was considered a differentially upregulated pathway and log2FC< −0.228 a differentially downregulated pathway. 

### 3.4. Consensus Cluster Plus Analysis

**Figure 5:**
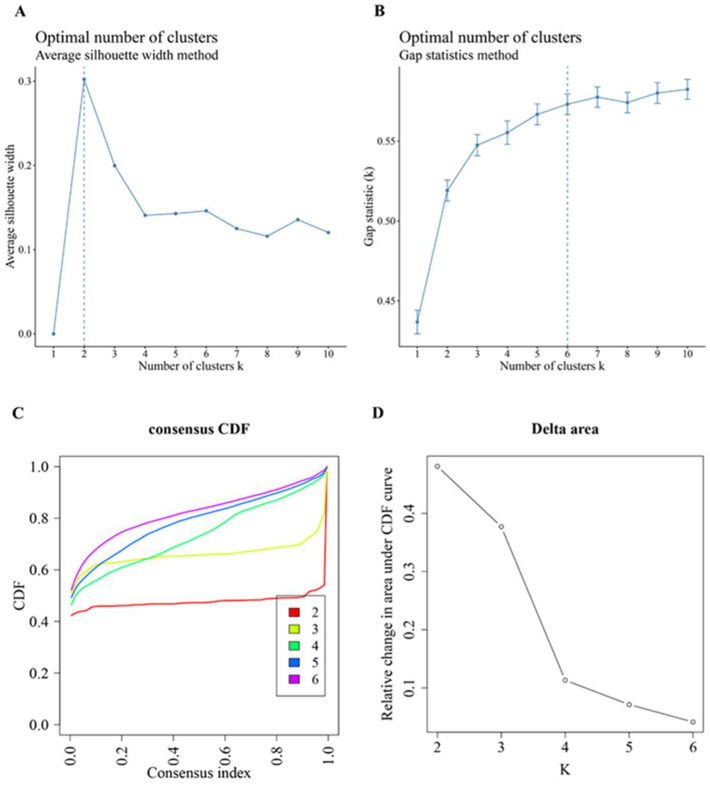
Consensus Cluster Plus analysis. (**A**,**B**) Average silhouette width and elbow method analysis results for PD & CKF significantly different pathway datasets; (**C**) Consensus cumulative distribution function (CDF) plot. This plot shows the cumulative distribution function of the scores when k takes different values. (**D**) Results of the relative change in area under the CDF curve for k and k-1 compared to the CDF curve. When k = 2 (since the data are not generally clustered into 1 class, there is no k = 1), the first point indicates the total area under the CDF curve at k = 2 (i.e., the area under the line in (**A**,**B**)), not the relative change in area. Choosing the final k value, the descending slope of the line in Figure 5C must be as small as possible, and the relative change in area under the CDF curve in Figure 5D for k and k-1 must be as small as possible compared to the area under the CDF curve.

In total, 19 significantly different intersecting pathways in PD and CKF were selected from case sample score matrices. Afterwards, the two sets of score matrices were merged and mean deweighted to obtain the PD&CKF significantly different pathway dataset composed of 19 pathways. Average silhouette width and elbow method results of the PD&CKF significantly different pathway dataset are given in Figure 5A,B. The results obtained show that the clusters that can be composed of PD case samples are groups 2 and 6, respectively (Figure 5A,B). Accordingly, for Consensus Cluster Plus analysis, the max k parameter was set to 6.

**Figure 6:**
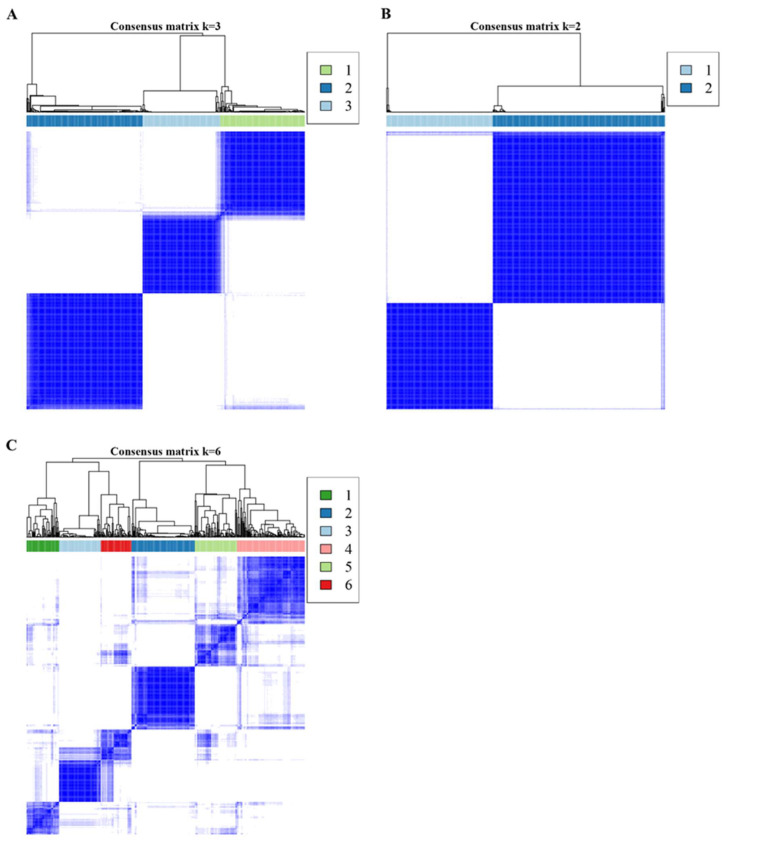
The clustering effect of Consensus Cluster obtained under different k values, displayed for 3 (**A**), 2 (**B**) and 6 (**C**).

**Figure 7:**
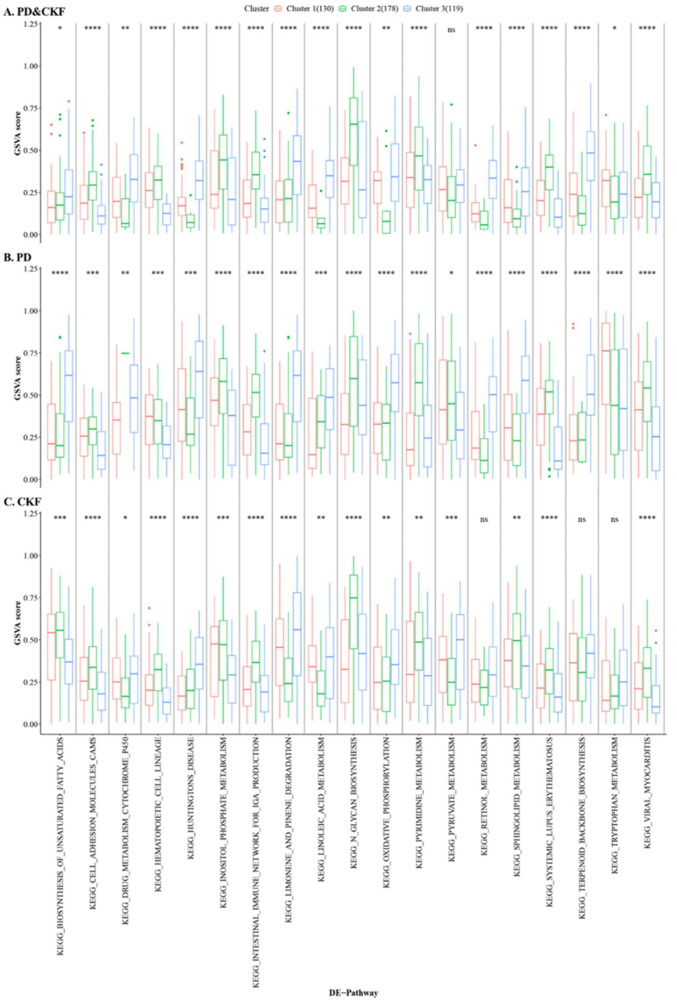
Cluster in the three access datasets of variability. The smaller the *p*-value of the test result, the more significant the sample difference result. “*” number corresponds to ns: *p* > 0.05, *: *p* ≤ 0.05, **: *p* ≤ 0.01, ***: *p* ≤ 0.001, and ****: *p* ≤ 0.0001.

In addition, the case sample of PD&CKF significantly different pathway dataset was divided into different clusters (Figure 5C,D) using Consensus Cluster Plus algorithm. The results obtained the best clustering when k = 3. Figure 6 shows the sample correlation results in the two datasets at k = 3 (Figure 6A), as well as at k = 2 and k = 6 (Figure 6B,C). For the three datasets of the PD & CKF significantly different pathway dataset, the PD significantly different pathway dataset, and the CKF significantly different pathway dataset, the Kruskal test was used to compare the difference of cluster in the three datasets (Figure 7A–C). Results were obtained for most pathways with significant differences between clusters.

### 3.5. Hub Cross-Talk Gene Screening

**Figure 8:**
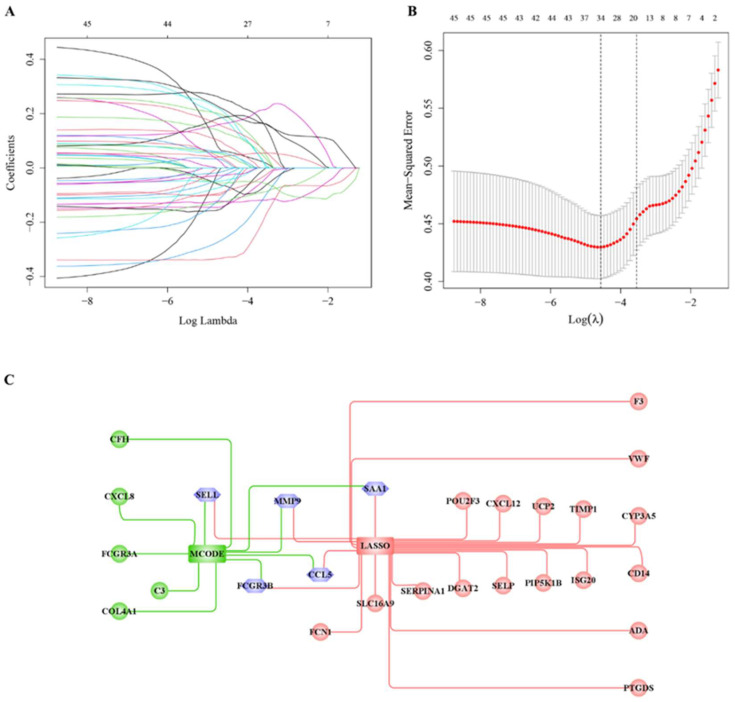
Hub cross-talk gene screening results. (**A**) Results of LASSO analysis; each line in the graph represents a gene, and the larger value of the horizontal coordinate (Log Lambda) when the gene tends to 0 indicates that the gene is more critical. (**B**) Results of model cross-validation. There are two dashed lines in the graph; one is the λ value lambda.min, when the mean square error is smallest, and the other is the λ value lambda.1se, when the mean square error is smallest, and either one of these two values can be chosen. (**C**) Intersecting genes in the hub genes (MCODE) and the 19 hub genes (LASSO).

Consensus Cluster Plus divided the case sample into three clusters. The expression values of 47 cross-talk genes in the three clusters were extracted and ANOVA analysis was performed. Figure 8A,B shows the expression values of these cross-talk genes using LASSO. According to the result of lambda.1se, 22 hub genes were revealed based on LASSO. Then 10 hub genes (MCODE) and 22 hub genes (LASSO) were extracted from the cross-talk genes (Figure 8C), whereby finally 5 hub cross-talk genes were obtained, i.e., *SELL*, *FCGR3B*, *SAA1*, *CCL5*, *MMP9*.

### 3.6. Prediction of Cluster Risk Based on Hub Cross-Talk Genes

**Figure 9:**
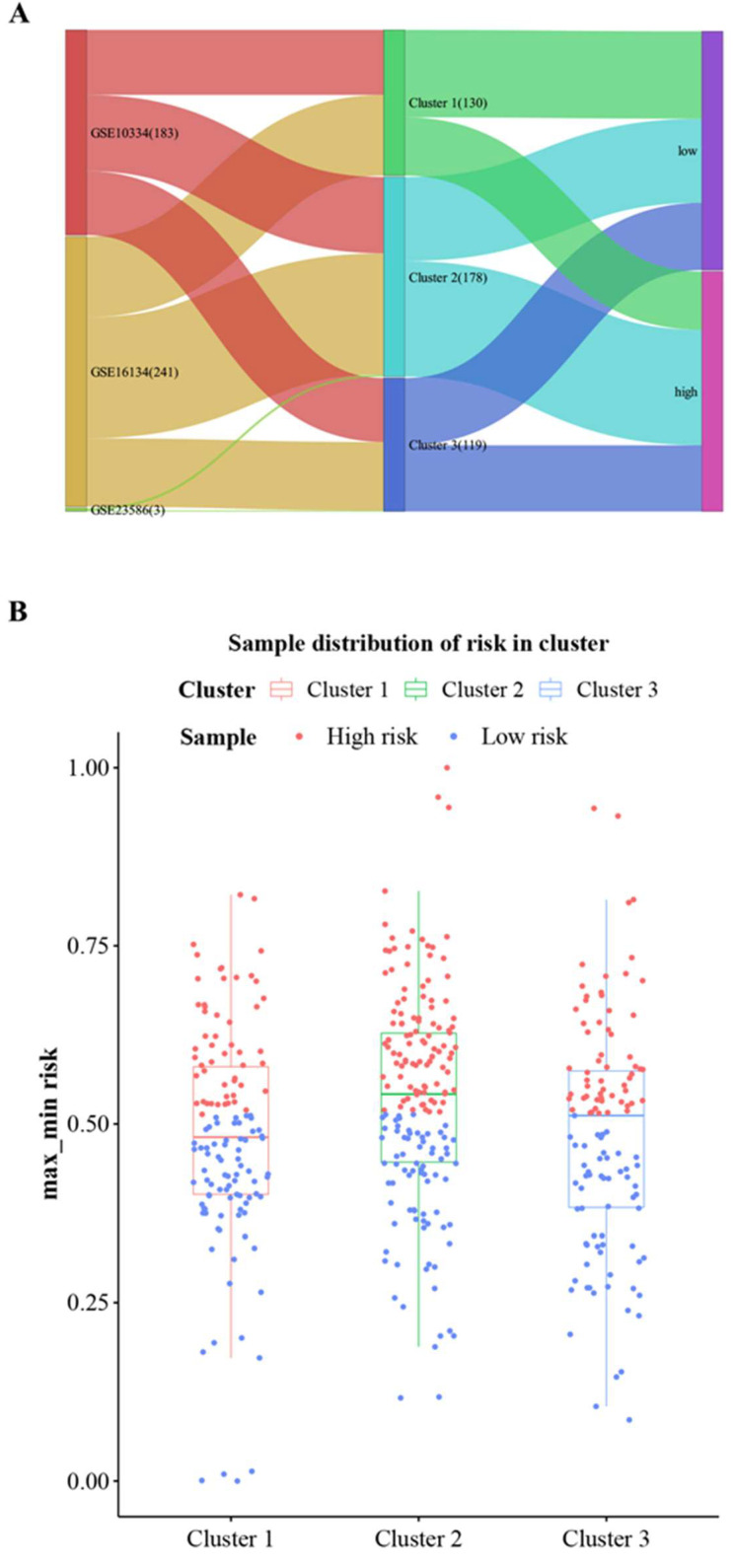
High- and low-risk enrichment results of disease samples in different clusters. (**A**) Distribution of samples in Series, Cluster, and Risk; (**B**) distribution of high-risk and low-risk samples in different clusters.

The regression coefficients of the five hub cross-talk genes were evaluated within LASSO regression analysis, which was the basis for calculation of the case sample risk score alongside with the hub cross-talk gene expression matrix. Then, the risk scores were min–max normalized and the samples were divided into high-risk and low-risk groups according to the median. Subsequently, the distribution of high- and low-risk samples was analyzed in Series, Cluster, and Risk (Figure 9A,B). The results found that the high-risk samples were mainly concentrated in Cluster2. This indicates that patients in Cluster2 are at higher risk than the other two groups.

### 3.7. Hub Cross-Talk Genes

**Figure 10:**
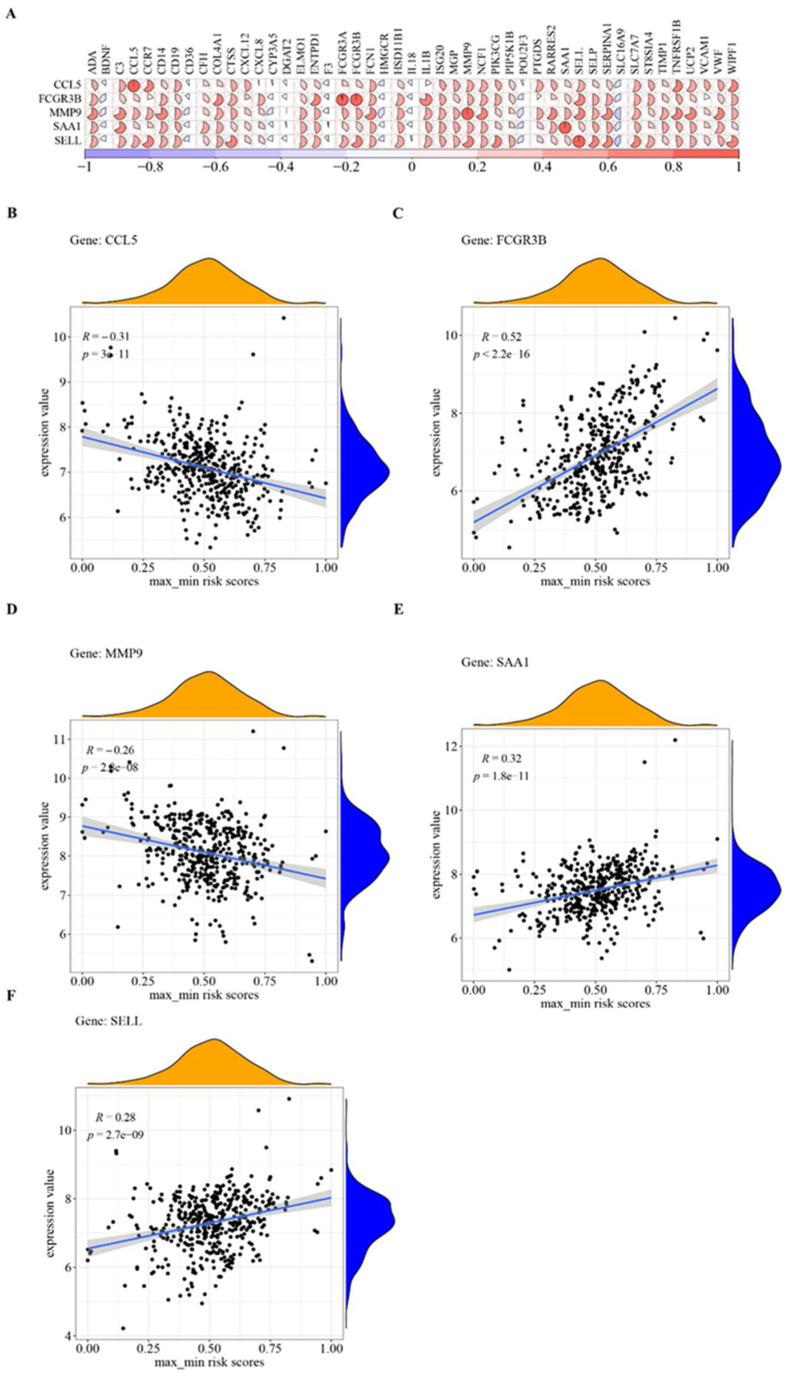
(**A**) Hub cross-talk gene and non-hub cross-talk gene correlations; (**B**–**F**) relationship between hub cross-talk genes and sample risk scores.

Pearson correlation coefficients were used to calculate the five hub cross-talk gene and 47 non-hub cross-talk gene correlations (Figure 10A). Furthermore, the relationship between the five hub cross-talk genes and the sample risk scores was analyzed, as presented in Figure 10B–F.

The results obtained were highly positive correlations between *FCGR3B* and *FCGR3A* (cor = 0.9671), *MMP9* and *CD14* (cor = 0.7384), *SELL* and *CTSS* (cor = 0.7207), and *SAA1* and *C3* (cor = 0.6743). *SELL*, *FCGR3B*, and *SAA1* were significantly negatively correlated with sample *SELL*; *FCGR3B* and *SAA1* were significantly positively correlated with sample risk; and *CCL5* and *MMP9* were significantly negatively correlated with sample risk.

**Figure 11:**
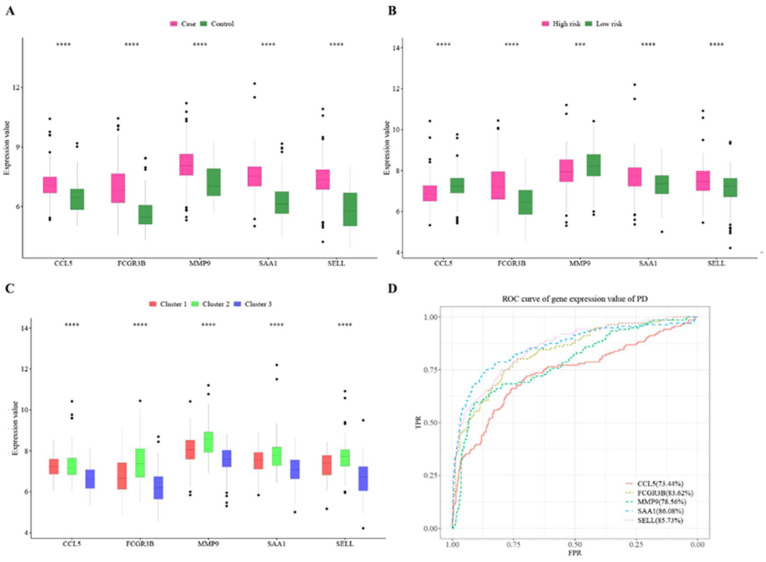
(**A**–**C**) Hub cross-talk gene expression levels in case–control cluster and high-risk vs. low-risk samples. The smaller the *p*-value of the test results, the more significant the sample difference results, while the more “*” on the graph, the *p*-value and “*” sign correspond to ***: *p* ≤ 0.001, ****: *p* ≤ 0.0001; (**D**) ROC assessment of hub cross-talk genes.

The expression levels of the five hub cross-talk genes were significantly higher in the case than in the control sample (Figure 11A). The expression levels of *CCL5* and *MMP9* were significantly lower in the high-risk sample than in the low-risk sample; the expression levels of *SELL*, *FCGR3B*, and *SAA1* were significantly higher in the high-risk sample than in the low-risk sample (Figure 11B). In addition, the gene expression levels of the five hub cross-talk genes were significantly higher in the Cluster2 sample than those in the Cluster1 sample and the Cluster3 sample (Figure 11C). The ROC assessment of the predictive effect of the expression values of the five hub cross-talk genes resulted in obtaining good predictions for all five genes (AUC > 70%) (Figure 11D).

### 3.8. Hub Gene *PPI* Network and Pathway Network

**Figure 12:**
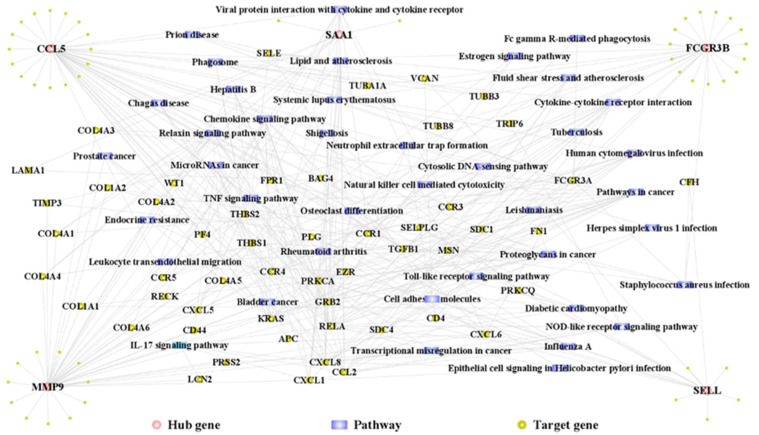
Hub cross-talk gene–pathway composite network. The network contains 146 nodes and 372 edges, which contain 5 hub genes, 101 target genes, and 40 pathways.

In the *PPI* network, 110 hub cross-talk gene–Target1 relationship pairs were revealed. A total of 7066 hub cross-talk gene–Pathway–Target2 relationship pairs were obtained based on the KEGG database. Furthermore, 305 Target1–hub cross-talk gene–pathway–Target1 closed-loop relationship pairs were obtained by screening genes in Target1 from the Target2 gene set. Based on the closed-loop relationship pairs, the complex network of hub cross-talk gene and pathway correlations was constructed (Figure 12). From the hub cross-talk gene–pathway network, it can be seen that these five hub cross-talk genes directly or indirectly regulate other genes and pathways related to periodontitis and kidney failure, thus affecting the process of both diseases.

## 4. Discussion

Main results: An overlap between periodontitis and CKF, including 47 potential cross-talk genes, was found, confirming the previously formulated hypothesis of shared genetic mechanisms. Different pathways were related to those genes, especially including microbiological (Lipopolysaccharide (LPS)-related processes), metabolic (e.g., AGE-RAGE, Lipids), cardiovascular, and inflammatory issues. Five hub cross-talk genes were identified, and they were *CCL5*, *FCGR3B*, *MMP-9*, *SAA1*, and *SELL*. Those genes had a good predictive value (AUC) and we’re associated with the respective disease status of the samples.

Comparison with literature and interpretation: 

The association between periodontitis and CKF has been explained in the literature and appears evident [3]. However, the potential causal interrelationship remains unclear. Figure 3 shows several potential pathways and processes which support a relationship between both diseases. One issue was the LPS-related response; LPSs are main virulence factors of potentially periodontal pathogenic bacteria, which can support the role of microbiota in the interrelation between periodontitis and CKF [5,8,10]. It has been already described that periodontal bacteria are able to enter the kidney tissues via the blood stream, inducing immunological reactions related to their virulence factors (LPS) [10]. Against this background, the current study’s findings appear plausible. Furthermore, processes and pathways of the immune response were related to the cross-talk genes in the current study, e.g., regulation of inflammatory response, leukocyte migration, or chemokine signaling pathway (Figure 3). The recent literature highlights the potential role of pro-inflammatory mediators along with oxidative stress in the interplay of periodontitis and CKF [5,8,16]. The elevation of systemic inflammation caused by periodontal diseases has been discussed previously, although it can be debated to what extent periodontal inflammation leads to systemic changes [2,17]. However, the increased (pro-)inflammation appears to be an important issue linking periodontitis and CKF, which will be discussed subsequently for the five hub cross-talk genes below. Finally, as shown in Figure 3, several metabolic issues might also link periodontitis and CKF, including, e.g., AGE-RAGE pathway (diabetes) or lipid and atherosclerosis. Those pathways could, on the one hand, be a hint of the relevant role of co-morbidities like diabetes mellitus as confounders for the association between periodontitis and CKF [4,5]. On the other hand, this might underline the systemic complexity of the relationship, as it is known that periodontitis increases the risk of atherosclerosis in patients with kidney diseases [11]. 

Due to the variety of results, further discussion will primarily focus on the five hub cross-talk genes. *CCL5*, also called RANTES, is a chemokine, which can be expressed by the majority of inflammatory cells, especially T-cells and monocytes [18]. Especially together with the G-protein-coupled receptor *CCR5*, *CCL5* is highly involved in a variety of immunological, inflammatory, and cancerous processes [18]. Thus, it is not surprising that *CCL5* has also been found to be related to periodontal inflammation [19,20,21]. In mice, *CCL5* was found to be involved in inflammatory bone resorption [21]. A Taiwanese study found *CCL5* single-nucleotide polymorphism to be related with aggressive periodontitis [19]. On the other side, *CCL5* was found to be related to chronic kidney diseases [22,23,24]. A study from Böger et al. (2005) showed that a polymorphism in the *CCL5* gene in diabetic patients with CKF was associated with mortality due to cardiac events [24]. This could be one explanation for the link between periodontitis and mortality in CKF patients, as described in the literature [12]. 

*FCGR3B*, which is one out of five genes encoding Fc γ receptors, is involved in recruiting neutrophils to the place of inflammatory reaction, as well as with the processing of immune complexes; therefore, this gene is of relevance in inflammation and (auto-)immunity [25]. Respective *FCGR3B* polymorphisms have been comprehensively studied in the context of periodontitis, whereby a meta-analysis showed that such a polymorphism is related with a nearly three-fold higher risk of developing an aggressive periodontitis [26]. Moreover, *FCGR3B* was found to be one key gene associated with oxidative stress in periodontitis [27], which could be of certain relevance in the interplay between periodontitis and CKF. *FCGR3B*, also known as CD16b, was also found to be related to kidney failure, especially with regard to antibody-triggered microcirculation inflammation after kidney transplantation [28,29]. Similar to *CCL5*, *FCGR3B* supports the link between periodontitis and CKF via increased pro-inflammation. 

The third potential hub cross-talk gene between periodontitis and CKF was *MMP-9*, a multidomain enzyme, which is excreted by neutrophils among other inflammatory cells, which is induced by many pro-inflammatory cytokines [30]. As matrix-metalloproteinases have been comprehensively investigated in the context of periodontal diseases, different studies are available highlighting the relevance of *MMP-9* in periodontal inflammation [31,32,33]. Moreover, a higher *MMP-9* concentration in serum and saliva has been found in patients with periodontitis and cardiovascular diseases [31]. Similarly, there is a high importance of *MMP-9* in CKF, especially due to its role in the degradation of the extracellular matrix [34]. Interestingly, *MMP-9* is also related to cardiovascular problems like atherosclerosis in CKF individuals [35]. This again points to two issues, on the one hand the relevance of increased inflammation in the interplay between periodontitis and CKF, and on the other hand, the potential link between periodontitis, CKF, and cardiovascular complications. 

The two genes with the highest predictive value in ROC analysis were *SAA1* and *SELL*. *SAA1*, i.e., serum amyloid A 1, is one of the most prominent members of acute phase response and therefore has high importance in primordial host defense [36]. An analysis of periodontal tissue samples revealed that *SAA1* would be one of the top 10 upregulated genes in periodontal diseased tissue [37]. Another study using a nonhuman primate model found that *SAA1* expression was associated with increased aging of gingival tissues, indicating a potential role in periodontal health and disease [38]. For CKF, it was found that *SAA1* concentration in serum of patients with kidney diseases was elevated [39]. As *SAA1* plays a role in renal recovery, several studies already used *SAA1* reprogrammed cells in an animal model [40,41]. As another aspect, proteomics research found elevated levels of *SAA1* in HDL of patients with CKF, indicating a potential relation with atherosclerosis [42,43]. As depicted in the regulatory network in Figure 12, *SAA1* was found to be connected with lipid and atherosclerosis, too. This again argues for the relationship of periodontitis–CKF–cardiovascular problems. 

Lastly, *SELL* encodes the protein L-selectin, which has been reported to play an important role in the initial leukocyte adhesion to the endothelial surface [44]. It is of relevance in inflammation as well as vascular pathogenesis, especially atherosclerosis [44]. Similar to *FCGR3B*, *SELL* was found to be a gene related to oxidative stress in periodontitis [39]. More than 20 years ago, studies pointed to the potential relevance of L-selectin in regulation of cell migration and cell adhesion, especially in severe forms of periodontitis [45,46]. On the other hand, *SELL* or l-selectin were found to be related with CKF [47,48,49]. A recent bioinformatics study found that upregulated expression of *SELL* would be associated with diabetic nephropathy [49]. Based on the bidirectional relationship between periodontitis and diabetes [50] and the potential relevance of diabetes-related pathways for the cross-talk genes in the current study (Figure 3 and Figure 12), this could be a further hint for the interrelation between periodontitis and CKF.

Taken together, the findings of the current bioinformatics study support an interaction of periodontitis and CKF. The primary connecting issue appears to be an increased degree of (pro-)inflammation, along with metabolic issues (e.g., diabetes) and vascular reaction (e.g., atherosclerosis). Those issues are somewhat unspecific, lacking a clear conclusion on causal mechanisms of action. This is similar to for other studies, e.g., the relationship between periodontitis and COPD [15]. As discussed in the previous study, the unspecific relationship between periodontitis and different chronic general diseases, like kidney, pulmonal, metabolic, or cardiovascular diseases, might indicate an underlying chronic systemic inflammatory syndrome [51]. This means that those diseases are different symptoms of one systemic underlying condition, indicating a co-incidence of the different diseases rather than a real causality. 

Strengths and limitations: This is the first comprehensive evaluation of the genetic cross-talk between periodontitis and CKF, with a reasonable methodical approach, which has been successfully applied previously for various different diseases [13,14,15]. The findings can form a basis for future research in the field; however, the results are restricted to this pre-experimental level. Therefore, the main limitation is that the findings are only on the transcriptomic level, without an experimental validation. Moreover, the study is limited by the inclusion of different datasets originating from different patients. Generally, different analytic approaches can be applied to examine the genetic overlap between two diseases. On the one hand, respective datasets of the two diseases can be assessed and analyzed separately as well as regarding shared differential expression [52]. This strategy requires the analysis of the different datasets from the two diseases, resulting in a certain heterogeneity of samples. Therefore, the current study has chosen the comparison between periodontitis datasets and CKF-related genes from DisGeNET. This platform is one of the largest collections of disease-related genes [53]. This allows the inclusion of a variety of valid disease-related genes, without a separate analysis of CKF datasets. This approach has already been used previously to compare oral and systemic diseases [54] and appears reasonable. However, the analytic approach of the current study did not consider another potentially relevant issue. Given that CKF can occur from a variety of different etiologies, performing analyses to investigate the relationship between periodontitis and specific subtypes of CKF (e.g., diabetic nephropathy, polycystic kidney disease) should be considered. This would extend the limits of this current study but might be a potentially relevant approach for further studies in the field and should therefore be considered in subsequent research projects. Considering those restrictions, the interpretation of the current results should stay on a hypothetical level, as it requires validation in animal models and clinical settings.

## 5. Conclusions

Periodontitis and CKF show genetic cross-talk, which is supported by five hub genes, i.e., *CCL5*, *FCGR3B*, *MMP-9*, *SAA1*, and *SELL*. In the interplay between periodontitis and CKF, primarily inflammation, along with metabolic and vascular issues, appear to be of relevance. Those findings require further validation.

## Figures and Tables

**Table 1:** Statistics of PD sample information and results of differential expression analysis.

	*Series*	*GSE10334*	*GSE16134*	*GSE23586*
Periodontitis (PD)	Platform	GPL570		
	Case sample	183	241	3
	Control sample	64	69	3
	Total sample	247	310	6
	*Limma analysis*			
	|Log FC|	|Log FC| > 0.5		
	*p*-value	*p*-value < 0.05		
	DEG-up	615	756	1873
	DEG-down	519	475	765
	DEG-Total	1134	1231	2638

**Table 2:** Results of variance analysis of 47 cross-talk genes.

Gene		Log FC			*p*-Value	
	*GSE10334*	*GSE16134*	*GSE23586*	*GSE10334*	*GSE16134*	*GSE23586*
***ADA***	6.50 × 10^−1^	7.46 × 10^−1^	1.33	7.69 × 10^−21^	2.39 × 10^−28^	3.20 × 10^−2^
***C3***	1.10	1.18	3.17	1.72 × 10^−24^	1.54 × 10^−33^	1.60 × 10^−3^
***CCL5***	6.04 × 10^−1^	6.47 × 10^−1^	1.61	7.55 × 10^−9^	5.75 × 10^−12^	4.68 × 10^−2^
***CCR7***	6.66 × 10^−1^	6.36 × 10^−1^	1.59	3.23 × 10^−9^	6.63 × 10^−10^	1.75 × 10^−2^
***CD14***	8.48 × 10^−1^	9.50 × 10^−1^	3.12	6.37 × 10^−14^	4.79 × 10^−21^	3.90 × 10^−2^
***CD19***	1.11	1.28	3.61	5.10 × 10^−23^	1.06 × 10^−29^	7.54 × 10^−3^
***CFH***	5.91 × 10^−1^	5.86 × 10^−1^	2.31	4.68 × 10^−13^	1.31 × 10^−15^	3.86 × 10^−2^
***COL4A1***	1.02	1.16	2.32	3.75 × 10^−21^	1.31 × 10^−31^	3.29 × 10^−2^
***CTSS***	8.01 × 10^−1^	8.91 × 10^−1^	1.63	4.92 × 10^−19^	6.29 × 10^−28^	3.68 × 10^−2^
***CXCL12***	9.27 × 10^−1^	1.05	2.60	1.76 × 10^−20^	1.42 × 10^−29^	4.14 × 10^−3^
***CXCL8***	1.27	1.20	2.91	3.76 × 10^−12^	7.13 × 10^−14^	4.62 × 10^−2^
***ELMO1***	8.16 × 10^−1^	9.14 × 10^−1^	1.02	1.96 × 10^−25^	6.66 × 10^−32^	4.81 × 10^−2^
***ENTPD1***	8.54 × 10^−1^	1.02	1.64	2.58 × 10^−23^	2.86 × 10^−33^	1.45 × 10^−2^
***FCGR3A***	1.09	1.21	3.78	3.00 × 10^−11^	4.97 × 10^−16^	2.14 × 10^−3^
***FCGR3B***	1.21	1.27	3.28	9.16 × 10^−16^	2.54 × 10^−20^	1.61 × 10^−3^
***FCN1***	1.00	1.08	1.29	2.30 × 10^−21^	8.58 × 10^−29^	3.49 × 10^−2^
***HSD11B1***	9.27 × 10^−1^	9.58 × 10^−1^	1.85	2.40 × 10^−22^	1.73 × 10^−29^	2.07 × 10^−2^
***IL1B***	1.05	1.01	1.92	2.04 × 10^−16^	3.57 × 10^−18^	1.19 × 10^−2^
***ISG20***	7.26 × 10^−1^	8.74 × 10^−1^	1.68	3.84 × 10^−10^	8.88 × 10^−15^	3.85 × 10^−2^
***MGP***	5.95 × 10^−1^	6.71 × 10^−1^	2.19	1.59 × 10^−13^	4.12 × 10^−20^	4.70 × 10^−4^
***MMP9***	8.56 × 10^−1^	9.19 × 10^−1^	2.46	5.94 × 10^−13^	1.00 × 10^−16^	9.14 × 10^−3^
***NCF1***	7.16 × 10^−1^	8.27 × 10^−1^	2.68	1.12 × 10^−15^	1.98 × 10^−20^	6.67 × 10^−3^
***PIK3CG***	5.58 × 10^−1^	6.23 × 10^−1^	2.73	2.18 × 10^−15^	2.07 × 10^−20^	2.67 × 10^−2^
***PIP5K1B***	6.23 × 10^−1^	7.19 × 10^−1^	1.68	6.77 × 10^−19^	6.56 × 10^−24^	6.55 × 10^−3^
***PTGDS***	8.20 × 10^−1^	8.89 × 10^−1^	1.93	7.83 × 10^−13^	1.82 × 10^−16^	1.26 × 10^−2^
***RARRES2***	8.46 × 10^−1^	9.29 × 10^−1^	2.12	1.44 × 10^−20^	9.84 × 10^−28^	9.29 × 10^−3^
***SAA1***	1.24	1.23	3.90	1.58 × 10^−23^	1.98 × 10^−29^	1.71 × 10^−2^
***SELL***	1.38	1.51	3.02	1.24 × 10^−23^	3.62 × 10^−33^	5.52 × 10^−4^
***SELP***	8.01 × 10^−1^	8.34 × 10^−1^	1.56	1.82 × 10^−26^	1.72 × 10^−35^	4.48 × 10^−2^
***SERPINA1***	5.78 × 10^−1^	5.85 × 10^−1^	1.42	2.20 × 10^−23^	4.20 × 10^−30^	3.61 × 10^−2^
***SLC7A7***	7.76 × 10^−1^	8.67 × 10^−1^	2.31	1.33 × 10^−18^	2.27 × 10^−24^	1.11 × 10^−2^
***ST8SIA4***	5.70 × 10^−1^	6.97 × 10^−1^	1.59	3.84 × 10^−16^	5.58 × 10^−23^	4.43 × 10^−2^
***TIMP1***	6.26 × 10^−1^	7.30 × 10^−1^	1.72	9.10 × 10^−9^	6.98 × 10^−13^	3.45 × 10^−2^
***TNFRSF1B***	5.51 × 10^−1^	6.22 × 10^−1^	1.50	7.83 × 10^−12^	1.30 × 10^−16^	1.30 × 10^−3^
***UCP2***	7.46 × 10^−1^	8.40 × 10^−1^	1.54	1.88 × 10^−14^	2.37 × 10^−20^	2.18 × 10^−2^
***VCAM1***	5.95 × 10^−1^	7.02 × 10^−1^	1.94	7.10 × 10^−9^	1.13 × 10^−13^	2.28 × 10^−2^
***VWF***	5.35 × 10^−1^	5.46 × 10^−1^	2.14	2.46 × 10^−23^	7.51 × 10^−27^	2.95 × 10^−2^
***WIPF1***	5.80 × 10^−1^	6.59 × 10^−1^	1.92	3.55 × 10^−21^	3.85 × 10^−30^	6.90 × 10^−3^
***BDNF***	−5.09 × 10^−1^	−5.87 × 10^−1^	−1.55	2.20 × 10^−18^	1.25 × 10^−28^	7.00 × 10^−3^
***CD36***	−6.54 × 10^−1^	−6.38 × 10^−1^	−1.42	2.57 × 10^−14^	4.02 × 10^−16^	6.56 × 10^−3^
***CYP3A5***	−8.03 × 10^−1^	−8.32 × 10^−1^	−1.29	3.17 × 10^−19^	3.92 × 10^−23^	3.45 × 10^−2^
***DGAT2***	−5.03 × 10^−1^	−5.23 × 10^−1^	−1.83	2.41 × 10^−17^	3.19 × 10^−21^	2.00 × 10^−2^
***F3***	−5.31 × 10^−1^	−5.50 × 10^−1^	−1.25	3.53 × 10^−9^	3.76 × 10^−11^	4.29 × 10^−2^
***HMGCR***	−8.73 × 10^−1^	−8.66 × 10^−1^	−1.92	9.71 × 10^−22^	6.44 × 10^−24^	4.90 × 10^−2^
***IL18***	−7.40 × 10^−1^	−7.46 × 10^−1^	−1.58	8.67 × 10^−15^	3.78 × 10^−16^	3.50 × 10^−2^
***POU2F3***	−5.59 × 10^−1^	−5.42 × 10^−1^	−2.51	8.94 × 10^−24^	1.25 × 10^−29^	1.09 × 10^−4^
***SLC16A9***	−1.12	−1.18	−3.33	4.46 × 10^−22^	1.91 × 10^−26^	1.32 × 10^−2^

**Table 3:** Analysis of topological properties of 45 cross-talk genes, where degree > 15 is tagged in Figure 3E.

Gene	Degree	ASPL	B C	C C	T C	Regulate
***VCAM1***	446	2.344697	0.770665	0.426494	0.004915	up
***NCF1***	49	3.736742	0.078767	0.267613	0.035714	up
***SERPINA1***	43	3.720644	0.08476	0.268771	0.051163	up
***C3***	37	3.535038	0.07269	0.282882	0.038038	up
***CCL5***	34	3.441288	0.065354	0.290589	0.039642	up
***PIK3CG***	33	3.628788	0.053927	0.275574	0.046832	up
***WIPF1***	33	3.543561	0.084819	0.282202	0.039627	up
***MMP9***	32	4.431818	0.052143	0.225641	0.043586	up
***CD14***	30	4.618371	0.055554	0.216527	0.033333	up
***HMGCR***	30	4.064394	0.05172	0.246039	0.055556	down
***TNFRSF1B***	29	4.044508	0.084218	0.247249	0.035632	up
***COL4A1***	29	4.441288	0.049476	0.22516	0.043478	up
***CD19***	25	3.616477	0.044886	0.276512	0.063333	up
***ADA***	21	3.657197	0.03425	0.273433	0.077922	up
***IL1B***	21	3.554924	0.049995	0.2813	0.068027	up
***PTGDS***	20	4.046402	0.029722	0.247133	0.075	up
***FCGR3B***	19	3.838068	0.029941	0.260548	0.068421	up
***UCP2***	19	4.025568	0.041251	0.248412	0.084211	up
***CD36***	19	3.679924	0.052201	0.271745	0.073227	down
***CFH***	18	3.405303	0.041513	0.29366	0.060847	up
***FCGR3A***	18	3.595644	0.036871	0.278114	0.07037	up
***VWF***	18	3.828598	0.031001	0.261192	0.079365	up
***ELMO1***	17	5.481061	0.028221	0.182446	0.117647	up
***BDNF***	17	3.839015	0.033603	0.260483	0.066176	down
***FCN1***	15	4.96875	0.023011	0.201258	0.066667	up
***SELL***	15	3.477273	0.028549	0.287582	0.088889	up
***HSD11B1***	14	4.594697	0.023016	0.217642	0.071429	up
***CXCL12***	10	3.697917	0.01378	0.270423	0.158333	up
***SELP***	10	4.05303	0.01282	0.246729	0.14	up
***TIMP1***	10	4.071023	0.014054	0.245639	0.1	up
***F3***	10	3.885417	0.017238	0.257373	0.116667	down
***CXCL8***	9	3.692235	0.014482	0.270839	0.117117	up
***IL18***	9	5.522727	0.013221	0.18107	0.222222	down
***SAA1***	8	4.737689	0.005895	0.211073	0.144531	up
***CTSS***	8	5.995265	0.01322	0.166798	0.125	up
***CCR7***	4	1	1	1	0	up
***SLC16A9***	4	1	1	1	0	down
***PIP5K1B***	3	5.605114	0.003786	0.178409	0.333333	up
***CYP3A5***	3	1	1	1	0	down
***SLC7A7***	2	1	1	1	0	up
***ST8SIA4***	2	5.831439	0.001894	0.171484	0.5	up
***DGAT2***	2	4.117424	6.53 × 10^−4^	0.24287	0.666667	down
***POU2F3***	2	1	1	1	0	down
***ENTPD1***	1	1	0	1	0	up
***MGP***	1	1	0	1	0	up

## Data Availability

The datasets used and/or analyzed during the current study are available from the corresponding author on reasonable request. Periodontitis data can be assessed from http://www.ncbi.nlm.nih.gov/database (assessed on 2 November 2022; *GSE10334*, *GSE16134*, *GSE23586*). Data for chronic kidney failure (CKF)-related genes were searched from DisGeNET https://www.disgenet.org/home/ (assessed on 4 November 2022).
